# Chemical Composition and Biological Activities of Essential Oils from Peels of Three *Citrus* Species

**DOI:** 10.3390/molecules25081890

**Published:** 2020-04-19

**Authors:** Lucia Caputo, Laura Cornara, Miriam Bazzicalupo, Clara De Francesco, Vincenzo De Feo, Domenico Trombetta, Antonella Smeriglio

**Affiliations:** 1 Department of Pharmacy, University of Salerno, via Giovanni Paolo II 132, 84084 Fisciano, Salerno, Italy; lcaputo@unisa.it; 2 Department for the Earth, Environment and Life Sciences, University of Genova, Corso Europa 26, 16132 Genova, Italy; cornara@dipteris.unige.it (L.C.); miriam.bazzicalupo@gmail.com (M.B.); 3 Department of Chemical, Biological, Pharmaceutical and Environmental Sciences, University of Messina, Viale Palatucci, 98168 Messina, Italy; clara904@hotmail.it (C.D.F.); dtrombetta@unime.it (D.T.); asmeriglio@unime.it (A.S.)

**Keywords:** *Citrus limon*, *Citrus × bergamia*, *Citrus × myrtifolia*, essential oil, phytotoxicity, eco-compatibility

## Abstract

Background: Fruit peels are generally underutilized byproducts of the food industry, although they are valuable sources of bioactive compounds. The aim of this study is to evaluate a new application for three *Citrus* peel EOs as bio-herbicides. Methods: After a micro-morphological evaluation of *Citrus* peels by SEM analysis, the phytochemical composition of the EOs of *Citrus × bergamia* Risso & Poit., *Citrus × myrtifolia* Raf., and *Citrus limon* (L.) Osbeck was characterized by GC/FID and GC/MS analyses. The in vitro phytotoxicity against germination and initial radical elongation of several crop and weed species was evaluated. Furthermore, the eco-compatibility of these EOs has been assessed by the brine shrimp (*Artemia salina*) lethality assay. Results: SEM analysis highlighted the morphometric differences of the schizolysigenous pockets among the peels of the three *Citrus* species. Oxygenated monoterpenes are the main constituents in *C. × bergamia* (51.09%), whereas monoterpene hydrocarbons represent the most abundant compounds in *C. × myrtifolia* (82.15%) and *C. limon* (80.33%) EOs. They showed marked and selective phytotoxic activity in vitro, often at very low concentration (0.1 μg/mL) against all plant species investigated, without showing any toxicity on *Artemia salina*, opening the perspective of their use as safe bio-herbicides.

## 1. Introduction

The *Citrus* genus belongs to the Rutaceae family and is characterized by fruits with distinctive aroma and delightful taste [1]. Currently, Italy is a leader in *Citrus* production, with about 3.0 million tons of fruit per year [2]. *Citrus* fruit peel shows many oil glands rich in essential oil (EO) but is an unexploited byproduct for juice production [3].

Natural byproducts represent a growing problem due to microbial spoilage of plant material; moreover, several factors such as their costs of drying, storage and shipment, limit further exploitation [4].

An example of byproducts are lemon [*Citrus limon* (L.) Osbeck] and chinotto peels (*Citrus × myrtifolia* Raf.), mostly used for the production of drinks, juices, and liqueurs. Instead, bergamot fruit peel [*Citrus × bergamia* Risso & Poit.] is used for the extraction of EO for food and cosmetic industry. Indeed, it gives to Earl Grey tea its distinctive flavor, is the base ingredient of cologne water (eau de cologne), and has various other perfumery uses.

Traditional medicinal uses of fruits belonging to different *Citrus* species have been reported in several countries, where peels or whole fruits are indicated to treat indigestion, skin inflammation and infections, muscle pain, cough and hypertension [5].

Bergamot is useful in relieving tension, relaxing spasms and improving digestion; the EO obtained from the fruit peel is used in aromatherapy for its sedative properties; in addition, it is also used in baths to treat vaginal infections [6].

Lemon fruit, due to its richness in vitamin C has been employed for a long time worldwide to treat infections and scurvy, while some reports also indicate uses for rheumatic conditions, and varicose veins [7]. The juice shows astringent, bactericide and anti-malaria properties [8], while the peel of the ripe fruit is known as carminative and stomachic [9].

The Chinotto fruit has been used as a primary ingredient of the Italian ‘Chinotto’ soft drink, with a bitter taste and digestive properties, and to a smaller extent, it is also used to prepare jams and candies, while its EO is appreciated in perfumery [10].

Beyond traditional uses, several studies showed various biological activities of *Citrus* EOs.

The anti-inflammatory activity of *C. × myrtifolia* and *C. × bergamia* has been reported [11,12], as well as the cytotoxic effects of the EOs derived from *C. × bergamia* [13]. The EO of *C. limon* has proved to be antimicrobial against Gram-positive and Gram-negative bacterial strains [14].

*Citrus* EOs could be used also for novel applications due to their richness in bioactive compounds [15], such as environmentally friendly antimicrobial agents, pest control drugs, and herbicides.

The use of chemical herbicides negatively affects human health and the environment; moreover, they are not actually appropriate for control use due to the developing resistance of some weeds [16].

For these reasons, great interest has been focused to finding alternatives to synthetic herbicides for agriculture [17], with a particular attention toward active compounds obtained from plants and their byproducts. Among these, EOs represent a rich potential source of alternative agents for weed control.

The aim of this study was to investigate and compare the micromorphology of flavedo oil glands of *C. limon*, *C. × bergamia*, and *C. × myrtifolia* and to elucidate the phytochemical profile of their EOs. Moreover, for the first time, the in vitro phytotoxicity of these EOs against germination and initial radical elongation of several easily germinable seeds such as *R. sativus* L., *L. sativa* L., *L. sativum* L., *S. lycopersicum* L., *L. multiflorum* Lam. and *P. oleracea* L., was evaluated.

Finally, the eco-compatibility of the EOs investigated was assessed by the brine shrimp (*Artemia salina*) lethality assay.

This allowed the evaluation, for the first time, of the activity of the *Citrus* EOs selected as new potential safe bio-herbicides.

## 2. Results and Discussion

### 2.1. Morphological Analyses

**Figure 1:**
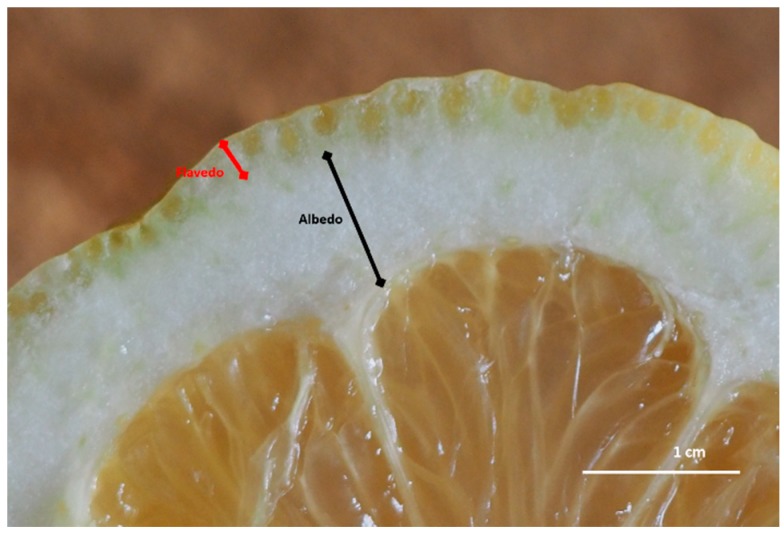
Cross section of *Citrus limon* peel. The exocarp is composed of two clearly distinguishable regions, the pigmented peripheral epicarp or flavedo, and the white middle layer called mesocarp or albedo. Enlarged oil glands are visible in the flavedo.

In the *Citrus* genus (Rutaceae), the fruit is an experidium and its exocarp forms the fruit peel, consisting of two regions. The epicarp or flavedo is the pigmented peripheral region containing enlarged oil glands, while the mesocarp or albedo is the white middle layer (Figure 1).

**Figure 2:**
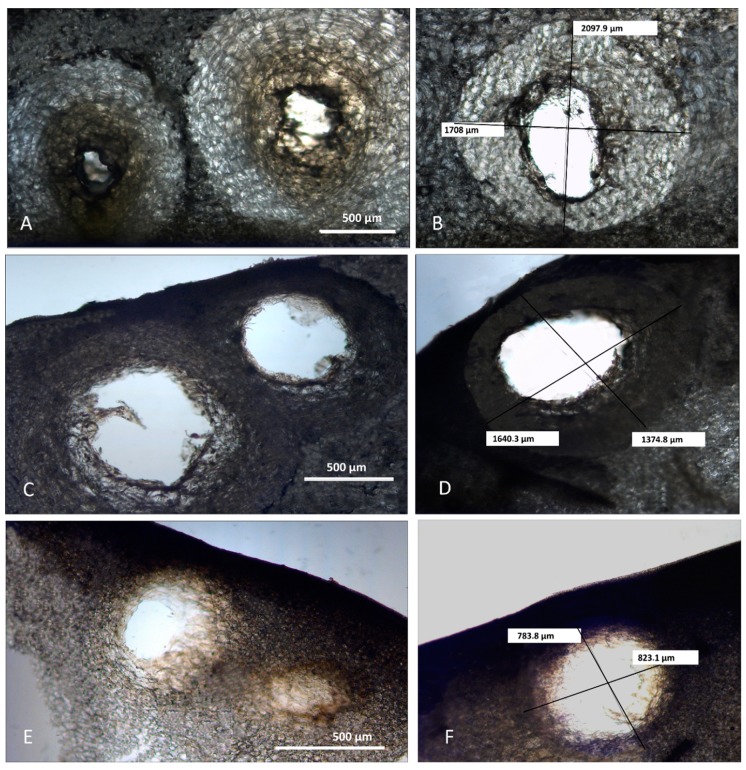
Light microscopy captures of oil glands from the peel of *C. limon*, *C. × bergamia*, and *C. × myrtifolia*, commonly known respectively as lemon, bergamot, and chinotto. Sub-prolate secretory cavities of *C. limon* (**A**,**B**). Oil glands sub-oblate to oblate-spheroidal in shape from the epicarp of *C. × bergamia* (**C**,**D**) and *C. × myrtifolia* (**E**,**F**). As shown by polar and equatorial diameter measures (**B**,**D**,**F**), lemon oil glands are larger and more oval with respect to those of bergamot and chinotto.

**Figure 3:**
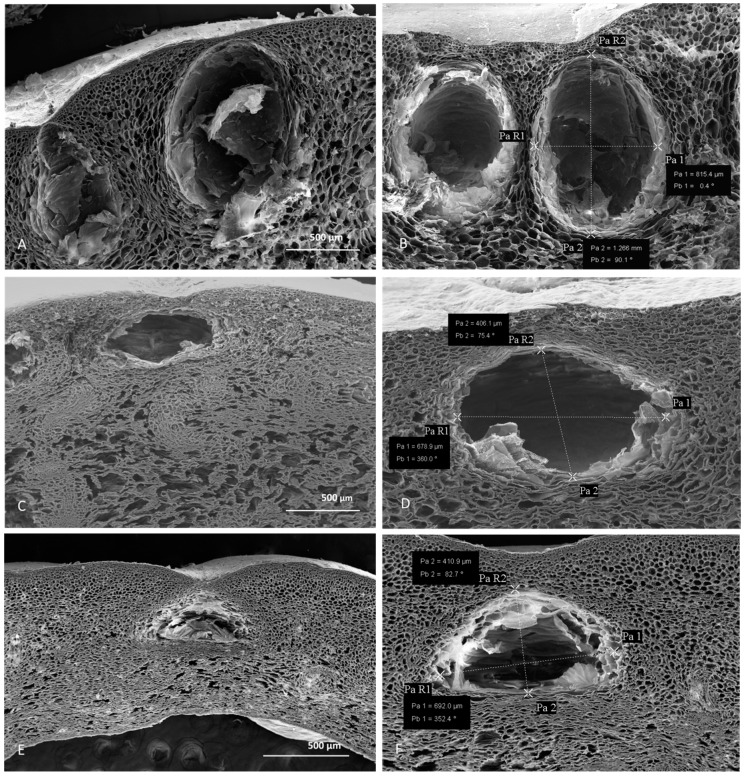
SEM micrographs of peel cross sections from *C. lemon* (**A**,**B**), *C. × bergamia* (**C**,**D**), and *C. × mytifolia* (**E**,**F**). Measures of the oil glands from the three different species are shown: lemon (**B**), bergamot (**D**) and chinotto (**F**).

Despite different opinions on the origin of the secretory structures in different *Citrus* species [18], several light and electron microscopic investigations revealed their schizolysigenous nature [19,20]. Previous studies also showed that within the fruit peels, a great variability in oil gland size and shape, was found. This could be due to different factors, such as the *Citrus* species that is considered, the stage of fruit development, and the timing of gland and secretory cavity formation [21,22]. In our study, to reduce the factors that affect oil gland variability among different species and within a single species, we analyzed oil glands from mature fruits along their maximum diameter. Light and scanning electron microscopy (SEM) analyses highlighted morphometric differences of schizolysigenous glands among the three *Citrus* species (Figure 2 and Figure 3; Table 1). In particular, mature oil glands of *C. limon* were generally subprolate-prolate in shape (Figure 2 and Figure 3A,B), while those of *C. × bergamia* (Figure 2 and Figure 3C,D) and *C. × myrtifolia* (Figure 2 and Figure 3E,F) were oblate-spheroidal.

In addition, the mean volume of the oil gland of *C. limon* was greater than those of *C. × bergamia* and *C*. *× myrtifolia* (Table 1). Our morphometric analyses are in agreement with previous data concerning other related species [22,23]. In addition, Voo et al. [22] also highlighted that the cavities of *C. × paradisii* continued to expand and to fill, reaching a regular oblate spheroid shape, so that cavity volume and oil accumulation correlate with later stages of fruit expansion. In our case, we only considered mature glands with a large central cavity, which were present in mature fruits. Our analyses show that, despite the larger volume of oil cavities found in *C. limon* peel, a greater yield of EO was instead obtained from *C. × bergamia*. We can therefore hypothesize that, in addition to oil cavity volume, other factors may influence the yield in EO, such as gland number/mm^2^, and different edaphic and climatic conditions of plant growth zones.

### 2.2. Phytochemical Analyses

*C. × bergamia, C. × myrtifolia,* and *C. limon* peel furnished pale yellow-green, yellow-orange, and pale yellow clear EOs, respectively. The yields, calculated on dry mass, were 1.20% for *C. limon,* 2.62% for *C. × bergamia,* and 0.60% for *C. × myrtifolia*, respectively.

Table 2 shows the phytochemical profile of the EOs, listing compounds according to their elution order on a HP-5MS column.

Oxygenated monoterpenes are the main constituents in *C. × bergamia* (51.09%) EO, whereas monoterpene hydrocarbons represent the most abundant compounds in *C. × myrtifolia* (82.15%) and *C. limon* (80.33%) EOs. Moreover, statistically significant differences were found in the phytochemical profile of the *Citrus* EOs investigated, which allows an easy differentiation between them (Table 2). Linalool (33.64%), linalyl acetate (9.22%) and α-terpineol (4.62%) were most abundant in the bergamot EO. The highest content of limonene (76.83%) was found in chinotto EO, whereas the most abundant amount of β-pinene (9.31%) and γ–terpinene (10.45%) was found in limon EO. Moreover, the latter was also the only one that showed a high amount of citronellol (8.19%).

Thirty-three components were identified in the EO of *C. × bergamia*, accounting for 100.00% of the total EO. Linalool (33.64%), limonene (32.29%), and linalyl-acetate (9.22%) are the main components, according to previous results about bergamot EO from Greece, although linalyl-acetate was the most abundant compound (30.33–40.51%) [24]. Other compounds, in lesser amounts, are γ–terpinene (6.39%), α-terpineol (4.62%), and β-pinene (4.29%). In our sample, the amounts of linalool and limonene were almost similar, although previous studies showed that limonene content was higher than linalool [25,26,27]. Moreover, the “essence degree”, defined as the linalool and linalyl acetate ratio [28] is 3.6, which is higher than in other bergamot EOs from Tunisia, Turkey, Algeria and Reggio Calabria (Italy) [25,26,27,28].

In the EO of *C. × myrtifolia*, 32 components were identified, accounting for 100.00% of the total EO. Limonene (76.83%), linalool (10.01%) and α-terpineol (2.66%) were the main compounds. This composition agrees in part with Plastina and co-workers [11], who reported limonene (54.3%) and linalyl acetate (22.9%) as the main constituents of this EO, although linalyl acetate is present in smaller amounts (1.00%).

The EO from *C. limon* showed the presence of 30 components, accounting for 97.89% of the total EO. Limonene (57.65%), γ-terpinene (10.45%), β-pinene (9.31%), and citronellol (8.19%) are the principal components. These results corroborate what was observed by Ghoorchibeigi and co-workers [29]; indeed, the EO of *C. limon* from Iran was rich in limonene (61.4%), γ-terpinene (11.3%), and β-pinene (13.1%). Limonene (60.34–70.81%), γ-terpinene (7.47–9.66%) and β-pinene (10.65–19.31%) were also the most abundant compounds in Sicilian limon EOs, with monoterpene hydrocarbons as the most abundant class (94.17–96.14%); citronellol instead was present in a lower amount (0.04–0.10%) [30]. Conversely, in the *C. limon* EO from Brazil, limonene was the main compound and geranial (7.89%), sabinene (7.51%), and neral (5.47%) are also present [31]; these compounds were absent in our sample.

### 2.3. Phytotoxic Activity

This is the first manuscript that investigates the phytotoxicity of these *Citrus spp.* EOs against germination and radical elongation of *R. sativus* L., *L. sativa* L., *L. sativum* L., *S. lycopersicum* L., *L. multiflorum* Lam., and *P. oleracea* L.

**Figure 4:**
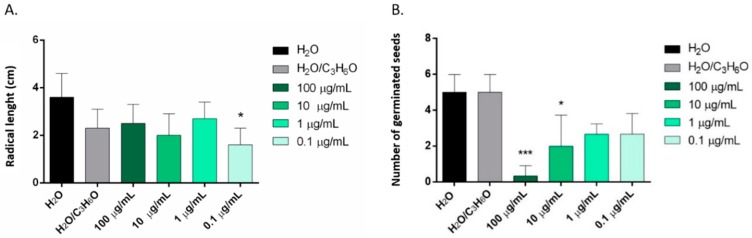
Phytotoxic activity of *C. × myrtifolia* EO against radical elongation of *L. multiflorum* (**A**) and germination of *P. oleracea* (**B**), 120 h after sowing. Results are the mean of three experiments ± standard deviation. * *p* < 0.05, *** *p <* 0.001 compared with control (ANOVA followed by Dunnett’s multiple comparison test).

**Figure 5:**
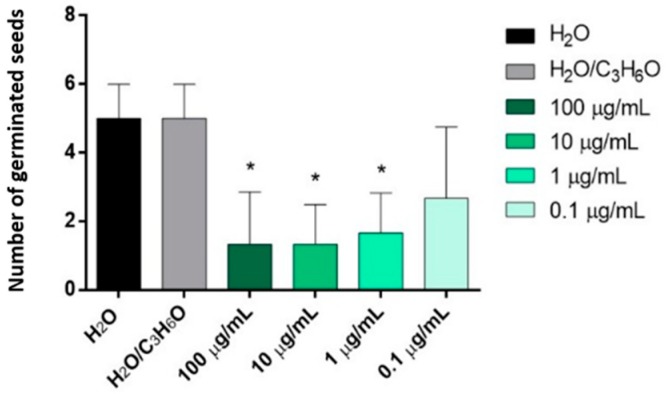
Phytotoxic activity of *C. × bergamia* (B) EOs against germination of *P. oleracea,* 120 h after sowing. Results are the mean of three experiments ± standard deviation. * *p* < 0.05 compared with control (ANOVA followed by Dunnett’s multiple comparison test).

*C. × myrtifolia* EO was able to inhibit the radical elongation of *L. multiflorum* seeds at the lowest concentration used (0.1 µg/mL) (Figure 4A). Instead, *C. × bergamia* and *C. limon* EOs showed no effect against radical elongation and germination of *L. multiflorum* (Table 3 and Table 4). Moreover, only *C. × myrtifolia* and *C. × bergamia* EOs were active against the germination of *P. oleracea*: the first at concentrations of 100 and 10 µg/mL (Figure 4B), whereas the second at concentrations of 100, 10, and 1 µg/mL (Figure 5). Instead, none of the EOs affected radicle elongation of *P. oleracea* (Table 3).

**Figure 6:**
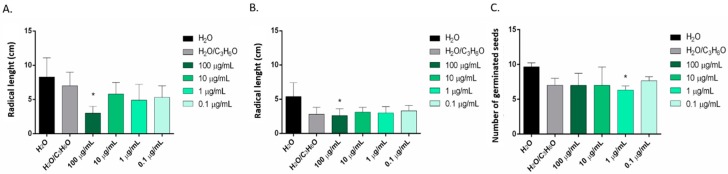
Phytotoxic activity of the EOs of *C. limon* against radical elongation of *S. lycopersium* (**A**), *L. sativum* (**B**) and germination of *R. sativus* (**C**), 120 h after sowing. Results are the mean of three experiments ± standard deviation. * *p* < 0.05 compared with control (ANOVA followed by Dunnett’s multiple comparison test).

Only the EO of *C. limon* seems to be effective against radical elongation of *S. lycopersium* and *L. sativum* at concentration of 100 µg/mL (Figure 6A,B) and against germination of *R. sativus* at a concentration of 1 µg/mL (Figure 6C). *C. × bergamia* and *C. × myrtifolia* showed no phytotoxic effects against *S. lycopersium*, *L. sativum* and *R. sativus* seeds (Table 3 and Table 4).

Finally, none of the EOs was active against germination or radicle elongation of *L. sativa* seeds (Table 3 and Table 4).

The phytotoxicity of *C. × myrtifolia* and *C. × bergamia* EOs has not been investigated before, and only a few literature reports were focused on the phytotoxicity of *C. limon* EO and of the other species belonging to the *Citrus* genus. *C. aurantiifolia* EO significantly reduced the germination of three monocot weeds: *Avena fatua*, *Echinochloa crus-galli* and *Phalaris minor*, at concentrations ranging from 0.10–1.50 mg/mL [32]. *C. limon* and *C. sinensis* EOs, from Algeria, did not exhibit a potent phytotoxic effect on two varieties of *Triticum aestivum* [33].

Instead, according to our results, Blazquez and co-workers [34] demonstrated that the peel EO of *C. limon* did not show any effect against *P. oleracea* seed germination, whereas Rolli et al. showed that *C. limon* EO reduced the length of the root and hypocotyle of *S. lycopersicum*, according to our results [35].

Hormesis is a dose-response relationship characterized by a biphasic effect, by which many organisms, exposed to a wide range of stimuli, show opposite behaviors depending on the dose [36]. Interestingly, in this study, *Citrus* EOs showed a hormetic dose response; in fact, after treatment of *R. sativus* with *C. limon* EO, there was a maximum response (less germination) in the low-dose zone. A similar effect was recorded on the radical length of *L. multiflorum* seeds after treatment with *C. × myrtifolia* EO.

Limonene is the main constituent of all EOs studied. In the literature, several studies have reported data about its possible phytotoxicity. This compound showed no significant effects against *Avena fatua*, *Echinochloa crus-galli*, *Phalaris minor* and *Zea mays* seeds [32,37]. However, limonene was weakly phytotoxic against *Alcea pallida* Waldst. & Kit., *Amaranthus retroflexus* L., *Centaurea salsotitialis* L., *Raphanus raphanistrum* L., *Rumex nepalensis* Spreng., *Sinapis arvensis* L. and *Sonchus oleraceus* L. [38]. A study to evaluate the phytotoxic effect of limonene against *Amaranthus viridis* L., was carried out. The compound showed a significant inhibition of the weed at very low doses (0.1 and 0.5 μL) [39].

Moreover, a previous study, observed that limonene was able to inhibit, in a significant way, the germination of *R. sativus* and radical elongation of *L. sativum* [40], according to our results. In light of this, it is possible to postulate that the phytotoxic activity observed for *C. limon* EO could be related to its main constituent, although a synergistic activity with other minor compounds cannot be excluded.

### 2.4. Brine Shrimp Lethality Assay

**Figure 7:**
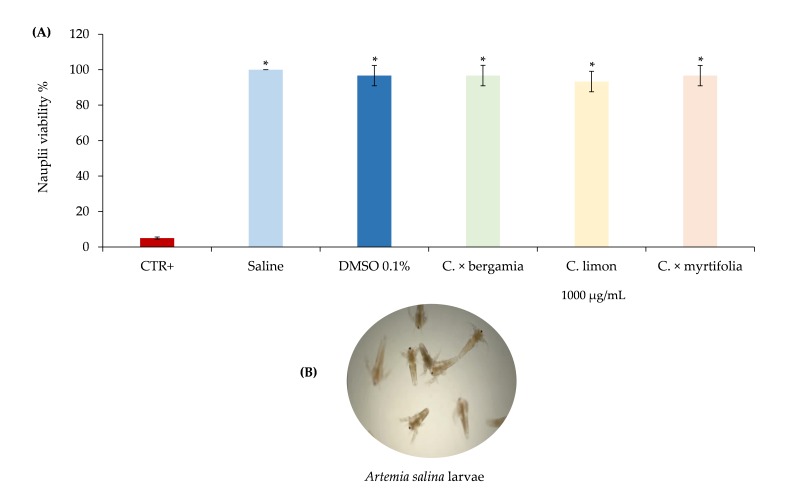
Brine shrimp lethality assay. Positive control (K_2_Cr_2_O_7_ 50 μg/mL), negative controls (saline and DMSO 0.1%) as well as the highest concentration (1000 μg/mL) of the *Citrus* EOs investigated were provided in panel (**A**). A representative photo of *Artemia salina* larvae (magnification 10×) in the plate well, acquired by a stereomicroscope equipped with a digital camera is reported in panel (**B**). Results are the mean of three independent experiments ± standard deviation.* *p* < 0.001 vs. CTR+ (ANOVA followed by Dunnett’s test).

The prospect of using *Citrus* EOs as new bio-herbicides requires the study of their eco-compatibility and in particular, the possible toxicity on aquatic organisms, since the potential use in agriculture would produce an inevitable passage of the same into underground aquifers. From this point of view, the toxicological test on *Artemia salina* is a useful, widely used, simple, rapid and inexpensive screening tool to evaluate the toxicity of the EOs [41]. Figure 7 shows the results at 24 h of brine shrimp lethality assay after treatment with *Citrus* EOs. Results at 48 h were perfectly comparable with the first ones (data not shown). Because no statistically significant difference between different concentrations of *Citrus* EOs was observed, only the highest concentrations in comparison with positive and negative controls were provided in Figure 7A.

No significant difference between the saline and solvent control was observed, indicating that DMSO 0.1% did not cause any visible effect to the exposed nauplii (mortality constantly below 4%) (Figure 7A). The positive control (K_2_Cr_2_O_7_ 50 μg/mL), showing an average mortality of 95% according to previous results [42], was statistically different (*p* < 0.001) from all other treatments (Figure 7A).

On the contrary, no statistically significant difference in the viability of nauplii treated with different concentrations (1000–0.01 μg/mL) of *C. limon*, *C. × myrtifolia,* and *C. × bergamia* EOs both at 24 and 48 h, was observed (data not shown).

Finally, no statistically significant difference, both in terms of swimming behavior and nauplii viability was recorded between negative controls and the highest concentration of *Citrus* EOs (Figure 7A). This is the first study that evaluates the toxicity of *C. limon*, *C. × myrtifolia,* and *C. × bergamia* EOs on *Artemia salina* larvae.

Since a much broader range of concentrations (1000–0.01 μg/mL) has been tested than that used for phytotoxicity tests (100–0.1 μg/mL), it is possible to speculate that the *Citrus* EOs tested are safe for aquatic organisms and, as such, biocompatible with regard to their potential use in agriculture as bio-herbicides.

Indeed, plant-derived natural phytotoxic compounds have been extensively studied in the recent years both for the growing evidence of their bioactivity and for the increasing concern about the possible toxicity of synthetic pesticides for humans and the environment.

Moreover, the use of agro-industrial byproducts can solve the costs of their disposal. The active molecules, furthermore, can be used as a promising template for standard bio-herbicides [43].

## 3. Materials and Methods

### 3.1. Chemicals

Alkane standard mix (C7-C40) and Na_2_SO_4_ were purchased from Sigma-Aldrich (Milan, Italy). Terpene standards were of analytical grade and were purchased from Extrasynthese (Genay, France). Dichloromethane was GC-grade and was purchased from Merck (Darmstadt, Germany).

### 3.2. Plant Materials and Essential Oil Extraction

Ripe fruits of *C. limon* (Limone Costa d’Amalfi IGP) were collected in November 2019, from biological orchards of the Amalfi coast (Salerno, Italy), while fruits of *C. × bergamia* (Fantastico variety) and *C. × myrtifolia* were harvested in November 2019 from biological orchards of the Ionian coast (Reggio Calabria, Italy) by local farmers and immediately sent to the laboratory. Prof. Vincenzo De Feo performed the taxonomic identification.

Fruits were peeled off manually by using a potato peeler and hydrodistilled by a Clevenger device [44], until no significant increase in the EOs volume was recorded (~3 h). EOs were dehydrated on Na_2_SO_4_ and stored in the dark with nitrogen headspace until use.

### 3.3. Micromorphological and Morphometric Analyses

Little portions of peel were removed from mature fruits along the maximum diameter, and then transversal sections of these portions were made by a razor blade, mounted in distillate water and observed under a transmission-light microscope (Leica D.M., 2000) equipped with a camera (DFC 320, Leica Microsystems, Wetzlar, Germany). Morphometric analyses were carried out for each species, by selecting 30–40 mature glands with a large central cavity, according to the gland development stages reported for *C. sinensis* [30]. The gland polar (PD) and equatorial (ED) diameters were measured by the Leica QWin, IM50 Image Manager Software. The gland cavity volume (V) was calculated by the formula: V = π × ED × 2 × PD/6, assuming a spheroid shape of cavities [31].

Samples for SEM analyses were obtained from pieces of about 1 cm^2^ excised from the mature fruit as above. They were fixed overnight with FineFIX solution (Milestone s.r.l., Bergamo, Italy) in 70% ethanol at 4 °C [45] and dehydrated through ethanol series: 80%, 90%, 95% and 100%, and critical point dried in CO_2_ (K850 CPD, 2M Strumenti S.r.l., Roma, Italy). After that, they were mounted on stubs, coated with 10 nm gold, and observed under SEM at 20 kV accelerating voltage (Vega3 LMU, Tescan Inc., Cranberry Twp, PA, USA).

### 3.4. Phytochemical Analysis

Analytical gas chromatography (GC) was performed by an Agilent gas chromatograph (7890A), equipped with a flame ionization detector (FID) (Agilent Technologies Santa Clara, CA, USA) equipped with a data handling processor. The separation was carried out using a HP-5MS capillary column (30 mm, 0.25 mm coated with 5% phenyl methyl silicone, 95% dimethyl polysiloxane, 0.25 μm film thickness) and helium as carrier gas (1 mL/min). One microliter of 10% EO/CH_2_Cl_2_ *v/v* solution was injected in split mode (50:1), setting the injector and detector temperature at 250 °C and 280 °C, respectively. Elution was carried out according to the following program: 60 °C for 6 min, increased to 270 °C at 3 °C/min, and held at 270 °C for 4 min [46].

Gas chromatography-mass spectrometry (GC-MS) analyses were carried out on the same instrument, coupled with a mass detector (5975C), with the same column and operative conditions used for the analytical GC but setting the ionization voltage to 70 eV, the electron multiplier to 900 V, and the ion source temperature to 230 °C.

EO components were identified by comparison of GC retention index (relative to C7-C40 n-alkanes), literature data [47], matching of mass spectral data with MS library NIST 08 [48], comparison of MS fragmentation patterns with those reported in literature, and co-injection with commercially available terpene standards. Quantification was carried out by extrapolation of the compound’s peak areas from GC-FID profiles.

### 3.5. Phytotoxic Activity

**Figure 8:**
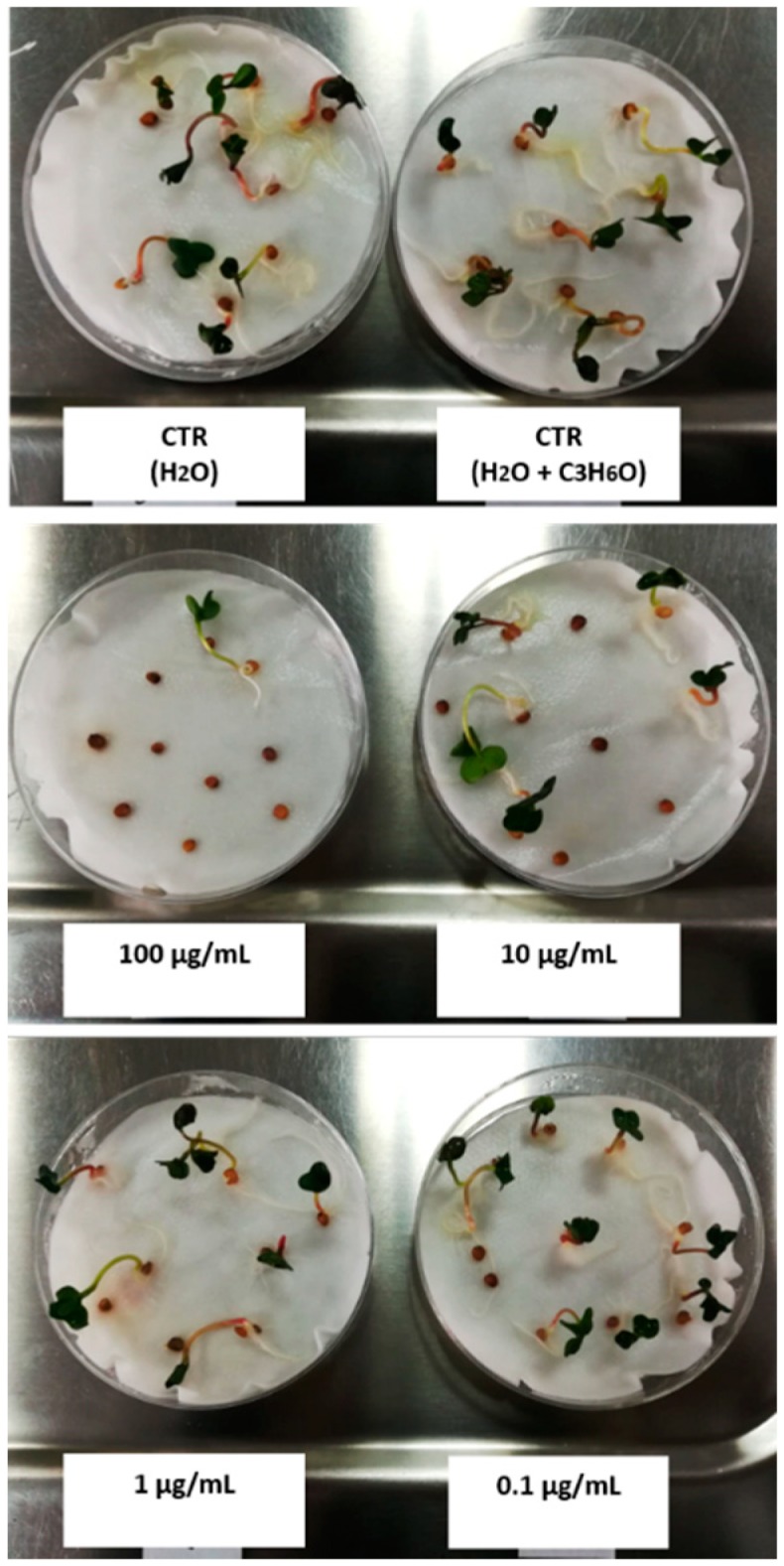
Representative photo of phytotoxic assay carried out on *R. sativus* seeds.

The phytotoxic activity was evaluated on germination and radical elongation of several species: *Raphanus sativus* L., *Lactuca sativa* L., *Lepidium sativum* L., *Solanum lycopersicum* L., *Lolium multiflorum* Lam., and *Portulaca oleracea* L. These seeds are often used for their easy and well-known germinability. A representative photo, in which it is possible to observe how different concentrations of EO can affect germination of *R. sativus*, was reported in Figure 8.

*R. sativus*, *L. sativa*, *L. sativum,* and *S. lycopersicum* seeds were purchased from Blumen group srl (Emilia Romagna). *L. multiflorum* seeds were purchased from Fratelli Ingegnoli Spa (Milano, Italy), and *P. oleracea* seeds from W. Legutko srl (Jutrosin, Poland). The seeds were sterilized in 95% ethanol for 15 s and sown in Petri dishes (Ø = 90 mm), on three layers of Whatman filter paper. They were impregnated with 7 mL of distilled water used as first control to verify the germinability of the seeds, 7 mL of water–acetone mixture (99.5:0.5, *v*/*v*) as second control because EOs were dissolved in this mixture for their apolarity, or 7 mL of the tested solution at different doses (100, 10, 1 and 0.1 µg/mL). Controls, carried out with water–acetone mixture alone, showed no appreciable differences in comparison with controls in water alone. The germination conditions were 20 ± 1 °C, with a natural photoperiod. Seed germination was observed in Petri dishes every 24 h. A seed was considered germinated when the protrusion of the root became evident [49]. On the fifth day (after 120 h), the effects on radicle elongation were measured in cm. Each determination was repeated three times, using Petri dishes containing 10 seeds each. Data are expressed as the mean ± standard deviation for both germination and radicle elongation.

### 3.6. Brine Shrimp Lethality Assay

*Artemia salina* eggs were purchased from a local aquarium pet shop. Citrus EO toxicity was evaluated according to Morabito and co-workers [50]. Dried cysts were placed in a hatcher chamber containing artificial seawater, and incubated for 2448 h at room temperature (RT). Natural ventilation and continuous illumination favored the larvae (nauplii) migration towards the opened centre of the chamber, where they hatched. Stock solutions 1000–0.01 mg/mL of each Citrus EO (*C. limon*, *C. × bergamia,* and *C. × myrtifolia*) in DMSO were prepared. Each concentration was seeded in a 24 well plate in triplicate and diluted 1:1000 *v/v* with artificial seawater (DMSO 0.1%). After that, 10 nauplii per well were added and incubated at RT for 48 h. Surviving nauplii without abnormal swimming behavior in each well were counted by a stereomicroscope (SMZ-171 Series, Motic) after 24 and 48 h. Three independent experiments in triplicates (*n* = 3) were carried out for each Citrus EO concentration. Two negative control groups (10 nauplii and artificial seawater added with DMSO 0.1%, and 10 nauplii and artificial seawater, respectively) as well as a positive control (K_2_Cr_2_O_7_ 50 μg/mL) [42], were also evaluated.

### 3.7. Statistical Analysis

Data were analyzed by one-way ANOVA, using Dunnett’s multiple comparisons test for phytotoxicity and brine shrimp lethality assay, and Tukey’s test for phytochemical characterization, by GraphPad Prism 6.0 software (GraphPad Software Inc., San Diego, CA, USA). Results were considered significant for *p* < 0.05.

## 4. Conclusions

The *Citrus* EOs analyzed showed marked and selective phytotoxic properties often at very low doses. However, EO phytotoxic effects depend not only on the dose and their chemical composition, but also on the sensitivity of the seeds tested. Crops are more tolerant than weeds to *C. × myrtifolia* and *C. × bergamia* EOs; in fact, the first EO selectively inhibits the radical elongation of *L. multiflorum* and germination of *P. oleracea* whereas the second one inhibits only germination of *P. oleracea.*

On the contrary, *C. limon* selectively inhibits the radical elongation of *L. sativum* and *S. lycopersium,* and the germination of *R. sativus*, being more active against crops than weeds.

None of the *Citrus* EOs tested showed toxicity on *Artemia salina,* with superimposable results, after observation at 24 and 48 h.

Considering the results, *C. × myrtifolia, C. × bergamia* and *C. limon* EOs merit further investigation for their potential use as new and safe bio-herbicides for crop control, to decrease the weed resistance and environmental pollution, and to increase simultaneously the people safety.

## Figures and Tables

**Table 1:** Morphometric measurements of oil glands from the peel of *C. limon, C. × bergamia* and *C. × myrtifolia.*

Species	Mean Polar Diameter (PD)	Mean Equatorial Diameter (ED)	Mean Volume (mm^3^)	Shape (PD/ED)
*C. limon*	1.73 ± 0.45	1.40 ± 0.38	2.20 ± 1.70	Prolate-spheroidal/sub-prolate/prolate (124.80 ± 13.10)
*C. × bergamia*	1.21 ± 0.35	1.31 ± 0.32	1.20 ± 0.90	Sub-oblate/oblate spheroidal (91.90 ± 9.00)
*C. × myrtifolia*	1.13 ± 0.28	1.25 ± 0.31	1.00 ± 0.70	Sub-oblate/oblate spheroidal (89.80 ± 9.40)

**Table 2:** Chemical composition of the *Citrus* essential oils investigated. Results are expressed as mean area percentage (%) ± standard deviation (S.D.) of three independent determinations in triplicate (*n* = 3).

Compound	*C. × bergamia*	*C. × myrtifolia*	*C. limon*	KI *^a^*	Identification *^b^*
α-Thujene	0.16 ± 0.01 *	-	-	924	1,2
α-Pinene	0.62 ± 0.04 *	0.34 ± 0.02 *	0.88 ± 0.04 *	939	1,2,3
Camphene	0.02 ± 0.01 *	-	t	954	1,2,3
β-Thujene	0.67 ± 0.05 *	-	-	968	1,2
Sabinene	-	0.18 ± 0.01 *	-	975	1,2
β-Pinene	4.29 ± 0.25 *	1.20 ± 0.03 *	9.31 ± 0.28 *	979	1,2,3
β-Myrcene	1.06 ± 0.08 *	1.70 ± 0.07 *	-	988	1,2,3
Octanal	-	0.44 ± 0.02 *	-	998	1,2
α-Phellandrene	0.06 ± 0.00	-	0.06 ± 0.00	1002	1,2
δ-3-Carene	0.75 ± 0.03 *	0.37 ± 0.02 ^§^	0.40 ± 0.02 ^§^	1008	1,2,3
α-Terpinene	0.20 ± 0.01 *	0.04 ± 0.00 *	-	1017	1,2,3
*p*-Cimene	0.13 ± 0.00 ^§^	-	0.25 ± 0.01 ^§^	1021	1,2
Limonene	32.29 ± 1.54 *	76.83 ± 2.44 *	57.65 ± 1.24 *	1024	1,2,3
1,8-Cineole	-	-	0.36 ± 0.02 *	1026	1,2,3
(Z)-β-Ocimene	0.25 ± 0.01 *	0.13 ± 0.01 *	t	1032	1,2
(E)-β-Ocimene	0.59 ± 0.03 *	0.72 ± 0.02 *	0.15 ± 0.01 *	1044	1,2
γ-Terpinene	6.39 ± 0.04 *	0.08 ± 0.00 *	10.45 ± 0.07 *	1054	1,2
*cis*-Sabinene hydrate	-	-	t	1065	1,2
Terpinolene	0.50 ± 0.02 *	0.16 ± 0.01 *	-	1086	1,2
*p*-Mentha-2,4(8)-diene	-	-	0.82 ± 0.03 *	1088	1,2
Linalool	33.64 ± 2.24 *	10.01 ± 0.55 *	0.76 ± 0.02 *	1095	1,2,3
(3Z)-Heptyl acetate	-	-	0.37 ± 0.01 *	1097	1,2
Dehydro-sabina ketone	-	-	0.07 ± 0.00 *	1120	1,2
*allo*-Ocimene	-	-	0.06 ± 0.00 *	1132	1,2
*cis*-Limonene oxide	-	-	t	1136	1,2
*trans-*Limonene oxide	-	-	0.17 ± 0.01 *	1142	1,2
Citronellal	-	0.01 ± 0.00 *	0.32 ± 0.02 *	1153	1,2
Isoborneol	-	-	0.11 ± 0.01 *	1160	1,2
(2E)-Nonen-1-al	-	-	0.07 ± 0.00 *	1161	1,2
*neoiso*-Isopulegol	-	-	1.14 ± 0.057 *	1167	1,2
Terpinen-4-ol	0.45 ± 0.02 *	0.26 ± 0.01 *	-	1174	1,2
α-Terpineol	4.62 ± 0.27 *	2.66 ± 0.08 ^§^	1.62 ± 0.10 ^§^	1186	1,2,3
Decanal	0.04 ± 0.00 *	0.10 ± 0.01 *	-	1201	1,2
n-Octyl acetate	0.11 ± 0.00 *	-	-	1213	1,2
Citronellol	-	-	8.19 ± 0.48 *	1225	1,2
Neral	0.35 ± 0.01	0.36 ± 0.02	-	1235	1,2
Carvone	-	0.12 ± 0.01 *	-	1243	1,2
Linalyl acetate	9.22 ± 0.34 *	1.00 ± 0.04 *	-	1254	1,2
Geranial	0.41 ± 0.01 ^§^	0.46 ± 0.02 ^§^	-	1264	1,2
Perillaldehyde	-	0.15 ± 0.01 *	-	1265	1,2
Bornyl acetate	0.02 ± 0.00 ^§^	0.01 ± 0.00 ^§^	-	1269	1,2,3
Nonyl acetate	0.02 ± 0.00	0.02 ± 0.00	-	1305	1,2
Citronellyl acetate	-	-	1.80 ± 0.08 *	1352	1,2
Neryl acetate	0.91 ± 0.05 *	0.39 ± 0.01 *	1.26 ± 0.02 *	1361	1,2
Neryl propionate	-	0.70 ± 0.03 *	-	1371	1,2
Neryl butyrate	1.47 ± 0.06 *	-	-	1393	1,2
(Z)-β-Caryophyllene	0.15 ± 0.01 *	0.04 ± 0.00 *	0.56 ± 0.01 *	1408	1,2,3
α-cis-Bergamotene	0.22 ± 0.01 *	-	0.77 ± 0.04 *	1411	1,2
(Z)-β-Farnesene	0.03 ± 0.00 ^§^	0.01 ± 0.00 ^§^	-	1440	1,2
γ-Muurolene	-	-	0.29 ± 0.02 *	1479	1,2
Germacrene D	0.03 ± 0.00 *	-	-	1484	1,2
Epi-Cubebol	-	0.02 ± 0.00 *	-	1493	1,2
Bicyclogermacrene	-	0.01 ± 0.00 *	-	1500	1,2
β-Bisabolene	0.02 ± 0.00 *	-	-	1505	1,2
(Z)-α-Bisabolene	0.31 ± 0.02 *	-	-	1507	1,2
(Z)-Nerolidol	-	0.05 ± 0.00 *	-	1532	1,2
Heneicosane	-	0.74 ± 0.04 *	-	2100	1,2
Total	100.00	100	97.89		
Monoterpene hydrocarbons	47.98	82.15	80.33		
Oxygenated monoterpenes	51.09	16.86	15.50		
Sesquiterpene hydrocarbons	0.76	0.08	1.62		
Oxygenated sesquiterpenes	0	0.07	0		
Others	0.17	0.84	0.44		

*^a^* Linear retention index on a HP-5MS column; *^b^* Identification method: 1 = linear retention index; 2 = identification based on the comparison of mass spectra; 3 = Co-injection with standard compounds; t = traces, less than 0.01%; * *p* < 0.001 vs. other *Citrus* EOs; ^§^ *p* < 0.05 vs. other *Citrus* EOs.

**Table 3:** Phytotoxic activity of the essential oils of *C. × bergamia*, *C. × myrtifolia*, *C. limon* against radicle elongation of *R. sativus*, *L. sativa*, *S. lycopersium*, *L. sativum*, *L. multiflorum*, and *P. oleracea* 120 h after sowing. Results are expressed as the mean of three experiments ± standard deviation.

	Radicle Elongation (cm)					
	*R. sativus*	*L. sativa*	*S. lycopersium*	*L. sativum*	*L. multiflorum*	*P. oleracea*
***C. × bergamia***						
CTR (H_2_O)	2.9 ± 1.6	4.1 ± 1.0	7.7 ± 2.4	3.8 ± 0.8	3.5 ± 0.9	2.8 ± 0.8
CTR (H_2_O + C_3_H_6_O)	1.8 ± 0.7	3.9 ± 0.6	4.2 ± 1.9	4.0 ± 0.9	2.8 ± 0.8	2.2 ± 0.5
100 μg/mL	4.3 ± 1.7	3.6 ± 1.5	6.0 ± 2.1	4.3 ± 0.8	2.5 ± 0.7	2.3 ± 0.6
10 μg/mL	3.4 ± 1.5	3.8 ± 0.8	5.0 ± 2.1	3.3 ± 0.6	2.7 ± 0.7	2.6 ± 0.5
1 μg/mL	2.0 ± 1.3	3.6 ± 0.6	4.5 ± 2.8	3.2 ± 0.9	3.4 ± 0.7	2.7 ± 0.8
0.1 μg/mL	2.5 ± 1.2	3.5 ± 0.5	5.5 ± 2.4	2.5 ± 0.7	2.8 ± 1.1	2.2 ± 0.5
***C. × myrtifolia***						
CTR (H_2_O)	6.1 ± 2.1	4.1 ± 0.7	5.5 ± 2.7	3.9 ± 1.4	3.6 ± 1.0	2.8 ± 0.8
CTR (H_2_O + C_3_H_6_O)	2.8 ± 1.6	3.5 ± 0.6	5.4 ± 3.0	3.2 ± 0.8	2.3 ± 0.8	2.6 ± 0.5
100 μg/mL	4.0 ± 2.2	3.3 ± 0.5	5.3 ± 2.8	4.7 ± 1.4	2.5 ± 0.8	2.8 ± 0.8
10 μg/mL	3.7 ± 1.4	3.9 ± 0.8	5.3 ± 2.9	4.8 ± 1.4	2.0 ± 0.9	2.2 ± 0.5
1 μg/mL	4.2 ± 2.0	3.8 ± 0.8	5.2 ± 2.7	2.7 ± 0.6	2.6 ± 0.7	2.4 ± 0.6
0.1 μg/mL	3.3 ± 1.0	4.1 ± 0.8	5.8 ± 3.0	3.2 ± 0.9	1.7 ± 0.7 *	2.3 ± 0.2
***C. limon***						
CTR (H_2_O)	5.4 ± 2.2	2.3 ± 0.1	8.3 ± 2.8	5.4 ± 2.0	4.8 ± 1.0	2.5 ± 0.5
CTR (H_2_O + C_3_H_6_O)	2.7 ± 0.6	2.3 ± 0.5	7.0 ± 2.0	2.8 ± 1.0	3.8 ± 0.9	2.4 ± 0.7
100 μg/mL	2.6 ± 1.0	1.4 ± 0.9	3.0 ± 1.0 *	2.6 ± 1.0 *	3.3 ± 0.8	2.3 ± 0.5
10 μg/mL	6 ± 2.7	1.8 ± 0.9	5.8 ± 1.7	3.1 ± 0.7	3.2 ± 0.9	2.4 ± 0.6
1 μg/mL	3.6 ± 1.0	1.1 ± 0.2	4.9 ± 2.3	3.0 ± 0.9	3.1 ± 1.0	2.4 ± 0.6
0.1 μg/mL	3.0 ± 0.7	1.6 ± 0.7	5.3 ± 1.7	3.3 ± 0.8	4.2 ± 1.3	2.1 ± 0.7

* *p* < 0.05 compared with control.

**Table 4:** Phytotoxic activity of the essential oils of *C. × bergamia*, *C. × myrtifolia*, *C. limon* against germination of *R. sativus*, *L. sativa*, *S. lycopersium*, *L. sativum*, *L. multiflorum*, and *P. oleracea* 120 h after sowing. Results are expressed as the mean of three experiments ± standard deviation.

	Germinated Seeds					
	*R. sativus*	*L. sativa*	*S. lycopersium*	*L. sativum*	*L. multiflorum*	*P. oleracea*
***C. × bergamia***						
CTR (H_2_O)	5.6 ± 1.5	9.0 ± 0.3	6.0 ± 0.5	7.7 ± 0.6	6.3 ± 0.6	5.6 ± 0.4
CTR (H_2_O + C_3_H_6_O)	5.6 ± 1.1	8.8 ± 0.2	6.2 ± 0.6	7.7 ± 0.6	8.7 ± 0.6	5.2 ± 0.8
100 μg/mL	3.0 ± 0.0	9.0 ± 0.0	5.0 ± 1.0	8.0 ± 1.0	7.6 ± 0.6	1.3 ± 1.6 *
10 μg/mL	4.0 ± 1.7	8.9 ± 0.2	5.3 ± 2.0	8.5 ± 0.7	6.3 ± 0.5	1.5 ± 1.2 *
1 μg/mL	3.4 ± 1.5	9.2 ± 1.2	4.6 ± 1.5	9.3 ± 1.1	6.3 ± 1.1	1.8 ± 1.3 *
0.1 μg/mL	4.6 ± 1.1	9.3 ± 0.7	7.6 ± 0.5	9.6 ± 0.6	7.0 ± 1.7	2.5 ± 2.7
***C. × myrtifolia***						
CTR (H_2_O)	5.0 ± 1.0	9.0 ± 0.0	7.0 ± 1.3	9.7 ± 0.6	6.3 ± 1.5	5.0 ± 0.5
CTR (H_2_O+ C_3_H_6_O)	4.7 ± 1.0	9.7 ± 0.6	7.2 ± 1.5	8.6 ± 0.6	7.0 ± 1.0	5.2 ± 0.3
100 μg/mL	6.3 ± 2.5	10.0 ± 0.0	7.0 ± 2.0	7.7 ± 1.5	8.0 ± 1.7	0.4 ± 0.3 ***
10 μg/mL	4.7 ± 1.5	9.0 ± 0.0	5.0 ± 3.0	9.0 ± 1.0	7.3 ± 1.1	2.0 ± 1.8 *
1 μg/mL	6.3 ± 0.6	9.3 ± 1.1	6.7 ± 1.5	10.0 ± 0.0	8.0 ± 2.6	2.9 ± 0.4
0.1 μg/mL	5.7 ± 1.5	9.3 ± 0.6	6.0 ± 1.7	9.7 ± 0.6	7.7 ± 0.6	2.9 ± 1.4
***C. limon***						
CTR (H_2_O)	9.7 ± 0.6	8.8 ± 0.3	10.0 ± 0.0	8.0 ± 0.0	8.3 ± 1.1	9.0 ± 1.4
CTR (H_2_O+ C_3_H_6_O)	7.0 ± 1.0	9.0 ± 0.0	9.3 ± 0.6	7.3 ± 1.2	8.3 ± 0.6	9.6 ± 0.6
100 μg/mL	7.7 ± 2.1	8.7 ± 0.4	9.6 ± 0.6	7.3 ± 0.6	9.3 ± 1.1	9.0 ± 1.0
10 μg/mL	8.0 ± 1.7	9.0 ± 1.1	9.3 ± 1.1	8.3 ± 1.1	9.3 ± 0.6	9.7 ± 0.6
1 μg/mL	6.3 ± 0.6 *	9.1 ± 1.3	9.7 ± 0.6	7.3 ± 1.5	9.7 ± 0.6	9.7 ± 0.6
0.1 μg/mL	8.0 ± 0.0	10.0 ± 0.0	10.0 ± 0.0	7.0 ± 1.0	9.7 ± 0.6	9.3 ± 0.6

* *p* < 0.05 compared with control. *** *p* < 0.001 compared with control.

## References

[1] Liu Y.Q., Heying E., Tanumihardjo S.A. (2012). History, Global Distribution, and Nutritional Importance of *Citrus* Fruits. Compr. Rev. Food Sci. Food Saf..

[2] Italian Society for Horticultural Science (ISHS). http://www.soihs.it/public/HtSItaly_website%20version.pdf.

[3] Moshonas M.G., Shaw P.E. (1979). Composition of essence oil from overripe oranges. J. Agric. Food Chem..

[4] Schieber A., Stintzing F.C., Carle R. (2001). By-products of plant food processing as a source of functional compounds—Recent developments. Trends Food Sci. Technol..

[5] Lv X., Zhao S., Ning Z., Zeng H., Shu Y., Tao O., Xiao C., Lu C., Liu Y. (2015). Citrus fruits as a treasure trove of active natural metabolites that potentially provide benefits for human health. Chem. Cent. J..

[6] Bown D. (1995). Encyclopaedia of Herbs and Their Uses.

[7] Chevallier A. (1996). The Encyclopedia of Medicinal Plants.

[8] Grieve M. (1984). A Modern Herbal.

[9] Chopra R.N., Nayar S.L., Chopra I.C. (1986). Glossary of Indian Medicinal Plants (Including the Supplement).

[10] Protti M., Valle F., Poli F., Raggi M.A., Mercolini L. (2015). Bioactive molecules as authenticity markers of Italian Chinotto (*Citrus × myrtifolia*) fruits and beverages. J. Pharmaceut. Biomed. Anal..

[11] Plastina P., Apriantini A., Meijerink J., Witkamp R., Gabriele B., Fazio A. (2018). In Vitro Anti-Inflammatory and Radical Scavenging Properties of Chinotto (*Citrus myrtifolia* Raf.) Essential Oils. Nutrients.

[12] Navarra M., Mannucci C., Delbò M., Calapai G. (2015). *Citrus bergamia* essential oil: From basic research to clinical application. Front. Pharmacol..

[13] Menichini F., Tundis R., Loizzo M.R., Bonesi M., Provenzano E., Cindio B.D., Menichini F. (2010). In vitro photo-induced cytotoxic activity of *Citrus bergamia* and *C. medica* L. cv. Diamante peel essential oils and identified active coumarins. Pharm. Biol..

[14] Solórzano-Santos F., Miranda-Novales M.G. (2012). Essential oils from aromatic herbs as antimicrobial agents. Curr. Opin. Biotech..

[15] Palazzolo E., Laudicina V.A., Germanà M.A. (2013). Current and Potential Use of *Citrus* Essential Oils. Curr. Org. Chem..

[16] Macías F.A., Molinillo J.M., Galindo J.C., Varela R.M., Simonet A.M., Castellano D. (2001). The use of allelopathic studies in the search for natural herbicides. J. Crop. Prod..

[17] Carvalho F.P. (2017). Pesticides, environment, and food safety. Food Energy Secur..

[18] Turner G.W., Berry A.M., Gifford E.M. (1998). Schizogenous Secretory Cavities of *Citrus limon* (L.) Burm. F. and A Reevaluation of the Lysigenous Gland Concept. Int. J. Plant Sci..

[19] Rapisarda A., Caruso C., Iauk L., Ragusa S. (1996). Applicazione dell’analisi di immagine nello studio delle ghiandole oleifere dei frutti di alcune specie di *Citrus*. Essenze Deriv. Agrum..

[20] Bennici A., Tani C. (2004). Anatomical and ultrastructural study of the secretory cavity development of *Citrus sinensis* and *Citrus limon*: Evaluation of schizolysigenous ontogeny. Flora.

[21] Knight T.G., Klieber A., Sedgley M. (2001). The Relationship between Oil Gland and Fruit Development in Washington Navel Orange (*Citrus sinensis* L. Osbeck). Ann. Bot..

[22] Voo S.S., Grimes H.D., Lange B.M. (2012). Assessing the Biosynthetic Capabilities of Secretory Glands in Citrus Peel. Plant Physiol..

[23] Bartholomew E.T., Reed H.S., Webber H.J., Batchelor L.D. (1943). General morphology, histology and physiology. The Citrus Industry.

[24] Sawamura M., Onishi Y., Ikemoto J., Tu N.T.M., Phi N.T.L. (2006). Characteristic odour components of Bergamot (*Citrus bergamia* Risso) essential oil. Flavour Frag. J..

[25] Melliou E., Michaelakis A., Koliopoulos G., Skaltsounis A.L., Magiatis P. (2009). High quality bergamot oil from Greece: Chemical analysis using chiral gas chromatography and larvicidal activity against the West Nile virus vector. Molecules.

[26] Kirbaşlar F.G., Tavman A., Dülger B., Türker G. (2009). Antimicrobial activity of Turkish citrus peel oils. Pak. J. Bot..

[27] Nabiha B., Abdelfatteh E.O., Faten K., Hervé C., Mohamed C. (2010). Chemical composition of bergamot (*Citrus bergamia*Risso) essential oil obtained by hydrodistillation. J. Chem. Chem. Eng..

[28] Figoli A., Donato L., Carnevale R., Tundis R., Statti G.A., Menichini F., Drioli E. (2006). Bergamot essential oil extraction by pervaporation. Desalination.

[29] Ghoorchibeigi M.O.N.A., Larijani K., Azar P.A., Zare K., Mehregan I. (2017). Chemical composition and radical scavenging activity of *Citrus limon* peel essential oil. Orient. J. Chem..

[30] Dugo P., Ragonese C., Russo M., Sciarrone D., Santi L., Cotroneo A., Mondello L. (2010). Sicilian lemon oil: Composition of volatile and oxygen heterocyclic fractions and enantiomeric distribution of volatile components. J. Sep. Sci..

[31] Gomes M.S., .Cardoso M.D.G., Soares M.J., Batista L.R., Machado S.M.F., Andrade M.A., de Azeredo C.M.O., Valerio Resendo J.M., Rodrigues L. (2014). Use of essential oils of the genus *Citrus* as biocidal agents. Am. J. Plant Sci..

[32] Fagodia S.K., Singh H.P., Batish D.R., Kohli R.K. (2017). Phytotoxicity and cytotoxicity of *Citrus aurantiifolia* essential oil and its major constituents: Limonene and citral. Ind. Crop. Prod..

[33] Ben Miri Y., Arino A., Djenane D. (2018). Study of antifungal, anti-aflatoxigenic, antioxidant activity and phytotoxicity of Algerian *Citrus limon* var. Eureka and *Citrus sinensis* var. Valencia essential oils. Essent. Oil Bear. Plant.

[34] Blázquez M.A., Carbó E. (2015). Control of *Portulaca oleracea* by boldo and lemon essential oils in different soils. Ind. Crop. Prod..

[35] Rolli E., Marieschi M., Maietti S., Sacchetti G., Bruni R. (2014). Comparative phytotoxicity of 25 essential oils on pre-and post-emergence development of *Solanum lycopersicum* L.: A multivariate approach. Ind. Crop. Prod..

[36] Calabrese E.J. (2018). Hormesis: Path and progression to significance. Int. J. Mol. Sci..

[37] Abrahim D., Braguini W.L., Kelmer-Bracht A.M., Ishii-Iwamoto E.L. (2000). Effects of four monoterpenes on germination, primary root growth, and mitochondrial respiration of maize. J. Chem. Ecol..

[38] Azirak S., Karaman S. (2008). Allelopathic effect of some essential oils and components on germination of weed species. Acta. Agric. Scand..

[39] Vaid S., Batish D.R., Singh H.P., Kohli R.K. (2011). Phytotoxicity of limonene against *Amaranthus viridis* L.. Bioscan.

[40] De Martino L., Mancini E., Almeida L.F.R., De Feo V. (2010). The antigerminative activity of twenty-seven monoterpenes. Molecules.

[41] Radulović N.S., Genčić M.S., Stojanović N.M., Randjelović P.J., Stojanović-Radić Z.Z., Stojiljković N.I. (2017). Toxic essential oils. Part V: Behaviour modulating and toxic properties of thujones and thujone-containing essential oils of *Salvia officinalis* L., *Artemisia absinthium* L., *Thuja occidentalis* L. and *Tanacetum vulgare* L.. Food Chem. Toxicol..

[42] Cruzeiro C., Amaral S., Rocha E., Rocha M.J. (2017). Determination of 54 pesticides in waters of the Iberian Douro River estuary and risk assessment of environmentally relevant mixtures using theoretical approaches and Artemia salina and Daphnia magna bioassays. Ecotoxicol. Environ. Saf..

[43] Duke S.O., Dayan F.E., Romagni J.G., Rimando A.M. (2000). Natural products as sources of herbicides: Current status and future trends. Weed Res..

[44] Maisonneuve S.A., Conseil de l’Europe (1996). Pharmacopée Européenne 1.

[45] Chieco C., Rotondi A., Morrone L., Rapparini F., Baraldi R. (2013). An ethanol-based fixation method for anatomical and micro-morphological characterization of leaves of various tree species. Biotech. Histochem..

[46] Smeriglio A., Trombetta D., Cornara L., Valussi M., De Feo V., Caputo L. (2019). Characterization and Phytotoxicity Assessment of Essential Oils from Plant Byproducts. Molecules.

[47] Adams R.P. (2007). Identification of Essential Oil Components by Gas Chromatography/Mass Spectroscopy.

[48] (2008). NIST 08 Mass Spectral Library (NIST/EPA/NIH). www.nist.gov.

[49] Bewley J.D. (1997). Seed germination and dormancy. Plant Cell.

[50] Morabito G., Trombetta D., Singh Brajendra K., Prasad Ashok K., Parmar Virinder S., Naccari C., Mancari F., Saija A., Cristani M., Firuzi O. (2010). Antioxidant properties of 4-methylcoumarins in in vitro cell-free systems. Biochimie.

